# Transmission Jeopardy of Adenomatosis Polyposis Coli and Methylenetetrahydrofolate Reductase in Colorectal Cancer

**DOI:** 10.1155/2021/7010706

**Published:** 2021-12-10

**Authors:** Younis Mohd, Parvinder Kumar, Haripriya Kuchi Bhotla, Arun Meyyazhagan, Balamuralikrishnan Balasubramanian, Mithun Kumar Ramesh Kumar, Manikantan Pappusamy, Karthick Kumar Alagamuthu, Antonio Orlacchio, Sasikala Keshavarao, Palanisamy Sampathkumar, Vijaya Anand Arumugam

**Affiliations:** ^1^Medical Genetics and Epigenetics Laboratory, Department of Human Genetics and Molecular Biology, School of Life Sciences, Bharathiar University, 641046 Tamil Nadu, India; ^2^Department of Zoology, Jammu University, Jammu, 180006 Jammu and Kashmir, India; ^3^Institution of Human Genetics, Jammu University, Jammu, 180006 Jammu and Kashmir, India; ^4^Human Genetics Laboratory, Department of Zoology, School of Life Sciences, Bharathiar University, Coimbatore, 461046 Tamil Nadu, India; ^5^Department of Life Sciences, CHRIST (Deemed to be University), Bangalore 560029, India; ^6^Department of Food Science and Biotechnology, College of Life Science, Sejong University, Seoul 05006, Republic of Korea; ^7^Department of General Surgery, Mahatma Gandhi Medical College and Research Institute, Pillaiyarkuppam, 607403 Pondicherry, India; ^8^Department of Biotechnology, Selvamm Arts and Science College (Autonomous), Namakkal, Tamil Nadu 637003, India; ^9^Laboratorio di Neurogenetica, Centro Europeo di Ricerca sul Cervello (CERC), Istituto di Ricovero e Cura a Carattere Scientifico (IRCCS) Fondazione Santa Lucia, Rome, Italy; ^10^Dipartimento di Medicina e Chirurgia, Università di Perugia, Perugia, Italy; ^11^Department of Chemistry and Biosciences, SASTRA Deemed to be University, Kumbakonam Tamil Nadu 612001, India

## Abstract

Colorectal cancer (CRC) is one of the globally prevalent and virulent types of cancer with a distinct alteration in chromosomes. Often, any alterations in the adenomatosis polyposis coli (APC), a tumor suppressor gene, and methylenetetrahydrofolate reductase (MTHFR) gene are related to surmise colorectal cancer significantly. In this study, we have investigated chromosomal and gene variants to discern a new-fangled gene and its expression in the southern populations of India by primarily spotting the screened APC and MTHFR variants in CRC patients. An equal number of CRC patients and healthy control subjects (*n* = 65) were evaluated to observe a chromosomal alteration in the concerted and singular manner for APC and MTHFR genotypes using standard protocols. The increasing prognosis was observed in persons with higher alcoholism and smoking (*P* < 0.05) with frequent alterations in chromosomes 1, 5, 12, 13, 15, 17, 18, 21, and 22. The APC Asp 1822Val and MTHFR C677T genotypes provided significant results, while the variant alleles of this polymorphism were linked with an elevated risk of CRC. Chromosomal alterations can be the major cause in inducing carcinogenic outcomes in CRCs and can drive to extreme pathological states.

## 1. Introduction

Increased westernization and its cultural influence have exalted colorectal cancer (CRC) spread in industrialized countries [1]. Usually, the prevalence of CRC is majorly sporadic 65-80% with few familial 15-30% modes of spread, implicating the major involvement of shared genes and environmental factors [2]. About 5% of the pathology is due to the mutational inheritance of the salient CRC genes with other familial forms linked with the interactions between gene and environment [2, 3]. Worldwide intensive increase of cases (about two million) and mortality (about one million) was registered in 2018 [4], with the shooting of two to five times CRC rates in the urbanized countries when compared to developing countries, suggesting a contrasting variation assortment in risk factors and analytical practices [5, 6]. The influence of sedentary lifestyle and western diet patterns, like food rich in animal proteins and fats, are the presumptive causes of elevated CRC cases in the current scenario. However, there can be an interlink between these factors and genetic characters in the Asian populations [7–12]. CRC progresses from single crypt lesions “adenomatous polyps” to carcinomas [13, 14] by following varying stages of invasion, lymph node involvement, site, and migration characterized by tumor-node-metastasis classification [15, 16].

Almost all cancers are materialized with chromosome breaks, especially in CRCs [17]. The pathological propagation of carcinogenesis involves the downregulation of tumor suppressor genes and upregulation of oncogenes [18]. The chromosomes' gain and loss or rearrangements via mutations are indispensable for chromosomal stability [19, 20]. Previously, gains of chromosomes 8q, 17q, and 20q or losses of 8p and 17p or unbalanced chromosomal alterations are seen in cancer patients [21, 22].

Any alterations in the APC gene can propagate to initiate CRC, making it the hotspot for CRC. The gene is located in the 5q21 region of chromosome 5 and is linked to the cases of familial adenomatous polyposis [23]. Based on literature studies, any pathogenic mutations in APC result in truncated protein production, suggesting that further alterations can influence redevelopment [24]. In 1993, CRC patients showed a mutation cluster region (MCR) on exon 15 between 1286 and 1513 codons [25]. Thus, confirming any missense APC polymorphisms can trigger and influence the risk of CRC by altering the protein structure, function, or location and hence modifies the physiology of the cellular entity.

Based on epidemiological studies, a combination of diet with lower folate concentration and extensive alcoholism is associated with augmented colon cancer risks and its precursor, the adenomatous polyp [26, 27]. Similarly, comprehensive hypomethylation results in chromosomal instability, and hypermethylation at the promoter-specific region suppresses the transcription causing in-gene silencing followed by tumor suppressor gene functional deprivation [28, 29]. The methylation end products are 5-methyl THF, crucial plasma folate, and an intracellular enzyme-substrate, 5,l0-methylene THF. The 5-methyl THF aids in the synthesis of *de novo* methionine and further methylation in DNA by provision [30]. In neoplastic studies linked with the colon and rectum, many MTHFR polymorphisms have been reported to date as the replacement of C→T at 677 positions resulting in the conversion of alanine to valine.

Similarly, replacing A with C on exon 7 causes the conversion of alanine into glutamate protein [31]. The presence of homozygous MTHFR TT is associated with lowered CRC risk in several case-control studies. The shielding effect is related to ample folate intake through diet, age and gender, and the proximal or distal colon [32]. Contrary to the case studies, few circumstances suggest that the presence of MTHFR TT genotype can also spike up CRC peril [33]. Reduced activity of MTHFR affects the methylation process in DNA via reducing the folate availability [34]. This folate deficiency could plausibly lead to carcinogenesis because of jeopardized DNA synthesis and repair or initiating DNA global hypomethylation as a possible early event of carcinogenesis [35].

Our study comprises of determining chromosomal alterations (CAs) in the APC and MTHFR gene polymorphism using the standard protocols based on these findings. We have also tried to correlate genetic compensation with CRC susceptibility. The current study is mainly aimed at investigating APC and MTHFR genotype's role and their associated genes in the South Indian CRC patients via PCR-restriction fragment length polymorphism (PCR-RFLP). The surveillance of identical abnormalities in all cell karyotypes within a tumor provides a strong indication for a clonal beginning of the tumor. These CAs can serve as an important marker to state the malignant states in individual cells to observe CRC jeopardy.

## 2. Methodology

### 2.1. Subject Recruitment

In the present study, we collected 65 blood samples from male CRC patients. An equal number of normal and healthy individuals were selected as controls, including those who did not undergo any therapy involving chemicals or radiation of any type. Inclusion criteria include patients in the different stages of disease who were recruited for the study. The study was performed based on medical reports of the practitioner (oncologist). The subjects were interviewed personally, and an open questionnaire was directed towards them for getting the relevant clinical details of etiological factors such as age, status, gender, tumor size, diet, medical and drug history, infection, hemochromatosis, cirrhosis, history of alcohol, smoking status, area of residence, and family history. Therefore, the exclusion criteria include the patients having undergone chemotherapy or any medications and with any other disease. The study excludes patients below 20 years and patients with other medical conditions like inflammatory bowel disease, hemorrhoids, or infections. The recruited subjects, both healthy and CRC patients, were categorized into two groups depending on their ages (group I ≤ 50 years and group II > 50 years), with average patient age as 24 (43.87 ± 3.62) in group I and 41 (62.79 ± 6.79) in group II. The recruitment of the subjects was consecutive with controls based on age (±2 years relaxed) with the attainment of informed consent. The current study got its ethical approval from institutional human ethical clearance (Ethical Reference No. BUHEC-006/2018), and the declaration was followed throughout the study. The peripheral blood samples of patients and controls were collected in heparinised tubes to culture the leucocyte, followed by staining the obtained chromosomal preparations with Giemsa to view the G-bands, and the supplementary clinical records were collected by reviewing the medical reports and patient charts plus determining the stage of cancer using the AJCC. The tumor location was seen in the right side of the bowel system at the cecum, appendix, ascending colon, hepatic flexure, and transverse colon. The left part of the bowel includes the descending colon, sigmoid colon, sigmoid junction, and rectum as per the definition of ICD-C-2 codes. The tumor grades are further categorized as well-differentiated, moderately differentiated, and poorly differentiated.

### 2.2. Sample Collection

For the study, 0.7 mL of blood was drawn by vein puncture in two sterile tubes for various biochemical, cytogenetic, and molecular assays, i.e., the tubes with EDTA and heparin along with the tissue samples obtained within 10 min of resection. Tissue samples were primarily analyzed for two constituents, i.e., one for histopathological diagnosis. At the same time, the other part is used for staging and molecular analysis by storing it at -80°C by following the standard criteria for both analyses [36].

### 2.3. Chromosome Aberration Assay

Cytogenetic studies were carried out using techniques like conventional analysis of the chromosome (karyotyping) using Trypsin G-Banding. The leucocytes were cultured from peripheral blood as suggested by Moorhead et al. [37]. In short, we were adding 0.5 mL of whole blood in 4.5 mL RPMI 1640 medium consisting of 10% fetal bovine serum, 2 mM L-glutamine, 1% streptomycin-penicillin antibiotics, and 0.2 mL reagent-grade phytohemagglutinin incubated at 37°C. At the 71^st^ hour, the cultures are treated with 0.1 mg/mL colcemid to block cells at the mitosis stage. After the completion of 72 hours, the lymphocytes are harvested by centrifuging the whole content (800–1,000 rpm/7 min) followed by incubation of 20 minutes in the hypotonic solution (KCl 0.075 M) at 37°C to swell the cells after treating twice with fixative (methanol and acetic acid (3 : 1 vol/vol)). The cytological slides were prepared by placing two to three drops of the concentrated cell suspension on the ice-cold acetic acid (60%) wetted slides followed by drying in a hot plate at 56°C for 2 min. For the CA analysis, 100 complete metaphase spreads were evaluated using a microscope (100x), especially to identify the numeric and structural anomalies as per the norms of the International System for Human Cytogenetic Nomenclature (ISCN). These gathered data were first plotted in a master table followed by transferring in a computer file.

### 2.4. APC and MTHFR Genotyping

DNA from the 65 CRC and 65 healthy samples was isolated and assessed for the genotypic frequency using PCR-RFLP. The following primers were utilized to carry out the RFLP: MTHFR(C677T): rs1801133: “5-GAGGCTGACCTGAAGCACTTGA-3′, 5′ATGCCTTCACAAAGCGGAAGA-3” and APC(T1822A): rs459552: “5-TAT TGC GGA GTG CGG GTC-3′, 5′-TCG ACG AAC TCC CGA CGA-3.”

The primers mentioned above were utilized to amplify the exons of MTHFR and APC, and the following protocol was followed for the PCR reaction-denaturation cycle at 94°C for 4 min followed by 35 cycles for 1 min at 94°C, 1.5 min at 55°C, and 2 min at 72°C. The PCR products were electrophoresed on 1% agarose with EtBr to view it under ultraviolet light. Further, the PCR products are digested at 37°C for about 8 hrs to 12 hrs with MspJ1 and AspI restriction enzyme. After digestion, the products were visualized in 4% metaphor agarose gel with EtBr.

### 2.5. Statistical Analysis

The “*t*” test was used to identify the statistically significant differences between the groups for analyzing the frequencies of APC and MTHFR genotypes. To estimate the strength of association between the polymorphisms of the genotype alleles in both controls and patients, the odds ratios (OR) and confidence intervals (CI) were calculated [38]. Similarly, to assess the variation between the controls and patients, the mean and standard deviation were calculated through one-way ANOVA. This analysis was carried out through IBM-SPSS Statistics for Windows, version 20.0 (IBM Corp., Armonk, N.Y., USA).

## 3. Results

The study group consists of 130 subjects comprising of each 65 CRC patients and controls with the inclusion of the characteristics linked to lifestyle, site of a tumor, grade of tumor, family history, follow-up checkups, and clinical analysis. The study group was grouped into two groups based on age as group I ≤ 50 years and group II > 50 years. The average patient age for both groups was *n* = 24 (43.87 ± 3.62) and *n* = 41 (62.79 ± 6.79), respectively. The groups were further categorized into smokers *n* = 37 (56.92%), nonsmokers *n* = 28 (43.08%), alcoholics *n* = 43 (66.15%), and nonalcoholics *n* = 22 (33.85%). The demographic characterization involved age and lifestyle components consisting of smoking and alcoholism habits, the stage and grade of the tumor, and its vocational site and patient history. The controls were recruited corresponding to CRC patients with a relaxation of ±2 year of age.

**Figure 1:**
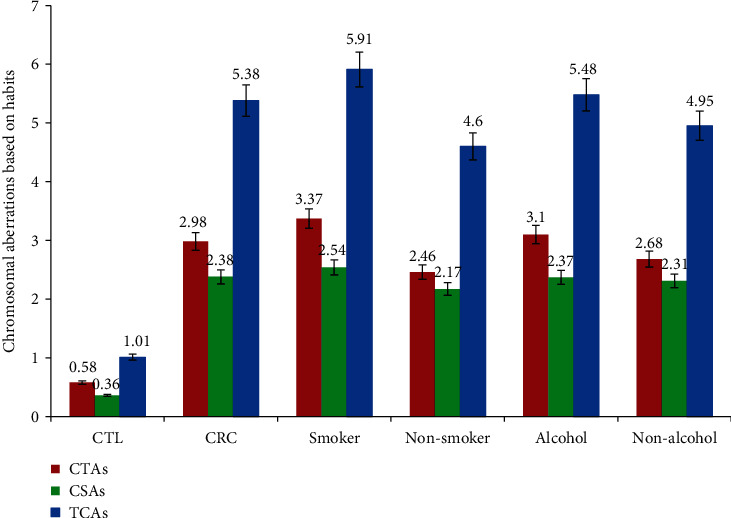
Chromosomal aberration frequency in CRC patients and controls based on their habits (smoker and alcohol).

Table 1 illustrates the chromosomal anomalies observed in the CRC patients to the controls. The CTAs of CRC subjects were recorded as 2.98 ± 1.11, which was significant while comparing with controls of their group (0.58 ± 0.55). Similarly, significant results were obtained while comparing the CSAs between the controls (0.36 ± 0.48) and the CRC subjects (2.38 ± 0.89). Even the CA mean values were found to be significant while comparing CRC subjects (5.38 ± 1.84) and controls (1.01 ± 0.85). All the CRC subjects showed significant values by ANOVA at *P* < 0.05. The following results were seen in the smoker CRC patients: CTAs-3.37 ± 1.06, CSAs-2.54 ± 0.90, and TCAs-5.91 ± 1.78; the following results were observed in the nonsmoker's group: CTAs-2.46 ± 0.96, CSAs-2.17 ± 0.86, and TCAs-4.60 ± 1.68; the alcoholic's group showed CTAs-3.10 ± 1.21, CSAs-3.10 ± 1.21, and TCAs-5.48 ± 1.99; the nonalcoholics had CTAs-2.68 ± 0.94, CSAs-2.31 ± 0.89, and TCAs-4.95 ± 1.67, respectively (Figure 1). The smokers and alcoholic group in the CRC patient showed statistically significant values (*P* < 0.05) for TCAs compared to their counter group nonsmokers and nonalcoholics by ANOVA.

**Figure 2:**
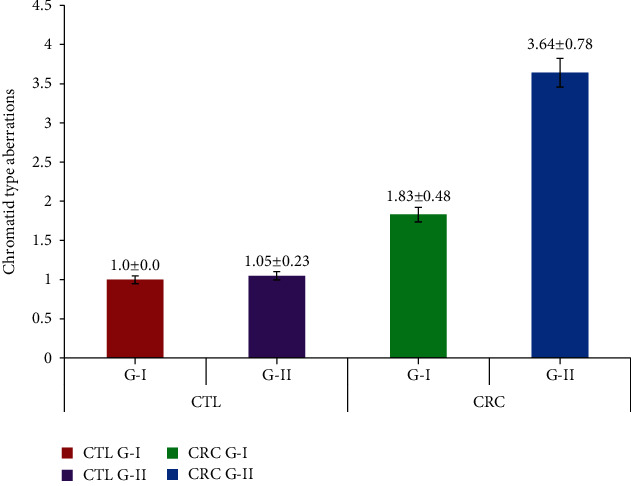
Chromatid type aberration frequency in CRC patients and controls based on their age.

**Figure 3:**
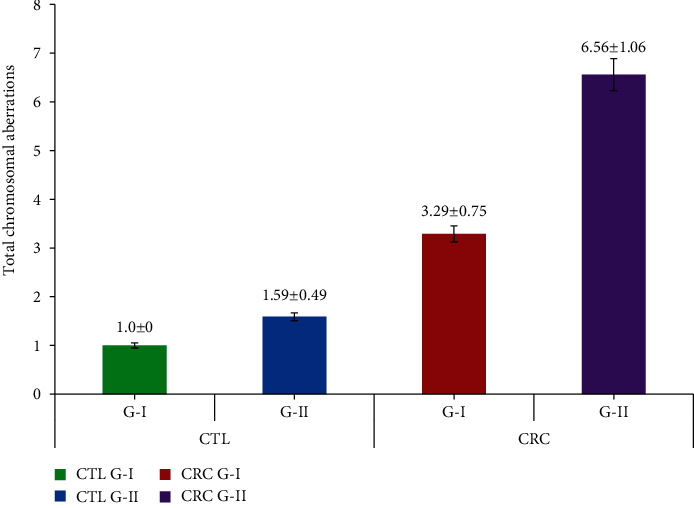
Chromosomal aberration frequency in CRC patients and controls based on their age.

**Figure 4:**
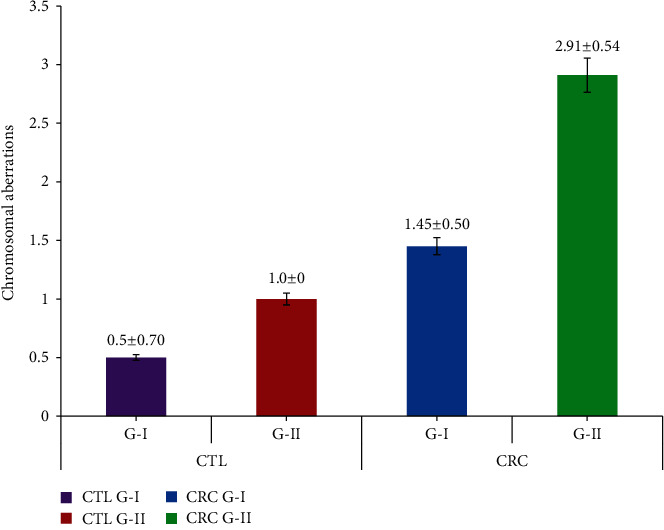
Total chromosomal aberration frequency in CRC patients and controls based on their age.

Table 2 elaborates the chromosomal damage observed in the CRC patients and controls. CTAs in group I and group II CRC subjects were 1.83 ± 0.48 and 3.64 ± 0.78, which were found to be significant when compared to their group I and II controls (1.00 ± 0.00 and 1.05 ± 0.23). The CSAs of group I and II CRC were 1.45 ± 0.50 and 2.91 ± 0.54, which were significant compared to their group I and II controls (0.50 ± 0.70 and 1.00 ± 0.00), respectively. The mean values of total TCAs in group I and II CRC were 3.29 ± 0.75 and 6.56 ± 1.06 and showed statistical significance when compared to their controls (1.00 ± 0.00 and 1.59 ± 0.49), respectively. All the CRC subjects showed significant values by ANOVA at the *P* < 0.05 level. The chromosomal alterations observed in Dukes stage A CRC patients were CTAs-1.75 ± 0.46, CASs-1.25 ± 0.46, and TCA-3.0 ± 0.75; Dukes stage B CTAs-2.0 ± 0.00, CASs-1.5 ± 0.53, and TCA-3.5 ± 0.53; Dukes stage C CTAs-3.5 ± 0.53, CASs-2.62 ± 0.91, and TCA-6.12 ± 1.24; and Dukes stage D CTAs-4.0 ± 0.75, CASs-3.12 ± 0.35, and TCA-7.12 ± 0.83, respectively (Figures 2–4). In CRC patients, the group II subjects, especially in Dukes stages C and D, showed statistically significant values in TCAs, compared to other groups at the *P* < 0.05 level by ANOVA.

Table 3 depicts the detailed karyotype results of CRC patients. The common anomalies observed were deletions at 5p, 9p, 13q, 16q, 19p, and 18q; duplication of 7, 8q, 12p, 14q, 16q, 20q, and 21q; inversion in 1q, 2p, and 4p; and translocation of 2, 11, and 22 chromosomes. A higher percentage of deletions were found as 46, XY, and del 5p.

**Figure 5:**
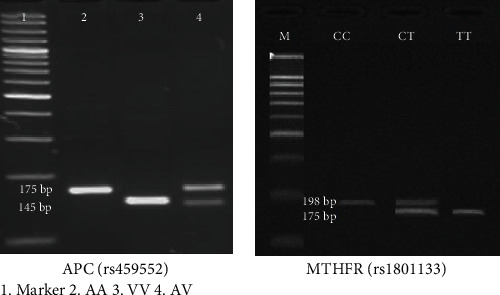
The 4% agarose gel image of the SNPs after the completion of RFLP-PCR of a sample to identify the genotypes in controls and other samples.

The genotype frequencies for the polymorphisms are reported in Table 4. Genotype distributions among control groups were in agreement with the Hardy-Weinberg equilibrium. The genotyping of the APCAsp1822Val genotypes was carried out in CRC patients and controls. APC Asp1822Val was categorized into three genotypes: AA, TA, and TT with the following frequencies of 58.46%, 33.84%, and 07.69% in CRC patients and 69.23%, 24.61%, and 06.15% in controls. Similarly, the genetic distribution of MTHFR 677 CC, CT, and TT genotypes was 63.07%, 27.29%, and 09.32% in CRC patients and 72.f30%, 23.07%, and 04.61% among controls. The genotype C/T of MTHFR polymorphism was associated with the risk of CRC, with a *P* value of 0.001.  Figure 5 illustrates the sample's agarose gel (4%) image for the selected SNPs after PCR-RFLP. After digestion, the presence of double bands represents the heterozygous genotype. The presence of a single brighter band correlates with homozygous dominant, and a less bright band corresponds to homozygous recessive.

## 4. Discussion

CRC is a typical menace where the cells lining the rectum's inner walls or colon alter and replicate faster without apoptosis. Usually, the genetic and environmental factors, including diet and lifestyle, trigger the carcinogenesis pathway in CRC [39]. A European prediction study assumed that the chronological transmutation and gene accumulation presented a new direction suggesting a 6.6% correlation of the mutational spectrum of a gene in large cohort groups of CRC. The heterogeneous prototype mutation implied the involvement of varied alternative genetic pathways in the pathogenesis and speculated a nonspecific genetic model in the tumorigenesis of the majority of CRC [40]. Usually, multiple abnormalities in the chromosome are observed in the case of CRCs. Literature studies associated with the pathways linked to distinct tumorigenesis have a significant correlation between a specific genetic pathway and differences in the Dukes (A to D) clinical staging outcomes among the CRC patients due to the involvement of various etiological factors in both the right and left CRCs [16, 41]. Genetic instability is considered a crucial indicator of both site and propagation of tumor [42]. Cytogenetic tools can detect each alteration, whether it is an initiation or a progression associated event due to causing gross chromosomal change [43], and these listed groups of alterations are in perfect agreement with our expression data for chromosome-specific trends, especially the presence of an alteration in chromosomes 1, 4, 5, 8, 13, 18, and 20. Our study observed increased CA frequencies among CRC patients compared to normal controls with a higher significance rate.

Similarly, CTAs and CSAs in both the groups of CRC subjects were significant to their controls with statistically significant results in the mean values of total aberrations in both groups regarding controls. Our study observed the following anomalies: deletions at 1p36, 5p, 17p, 18q, 18q22.q23, 21p, and 22p. A higher percentage of deletions were found in the 46, XY, and del 18p-. Other than deletion, we have also observed the translocations at the chromosomes 46, XY t(2; 11p), and t(2; 22), suggesting our findings as synonymous with the literature reports on CRC showing higher addition of chromosomes 7, 8q, 13q, and 20q and deletion of 4 and 18q [44, 45]. Epidemiological studies on sporadic CRC cases have highlighted the increased loss of heterozygosity (LOH) in the regions of chromosomes 5, 8, 11, 12, and 17 and predominantly at 14 and 18; these chromosomes were seen in cases of either whole chromosome or segmental uniparental while performing the array for the SNPs [46]. Genomic reorganizations are considered a crucial stage for cancer pathogenesis. They result in more frequent specific structural aberrations, resulting in loss of tumor suppressor gene function or increased oncogenic stimulation as seen in leukemia lymphomas and other solid tumors [47]. Our study primarily dealt with the presence of APC and MTHFR gene polymorphism in the South Indian population. Epidemiology studies have reported approximately 60% mutations in the APC gene in the codon region of 1286 and 1513 of exon 15 as a prime spot for CRC pathogenesis [48]. The Won et al. [49] study reported FAP patients to have about 61% of the mutational rate in the APC compared to Caucasians, which has a rate of 80% [50]. The frequency of the APC mutation was seen to be in between the previous literature studies in our work. The mutations in the APC gene were shown to have a significant positive correlation in terms of patient age as the APC mutations were seen less frequently in patients with age lesser than 50 (*P* = 0.040), and older males were illustrating higher APC mutational frequency. Based on the population genetics, the APC mutation incidence is reported as 37–56% in Europeans, 26–42% in Asians, and 60% in the US [51, 52].

Moreover, Iwamoto et al. [53] observed the functional loss of the APC gene in 83% of cases of colon cancers. The tumor commences by retaining APC integrity in the neoplasm as a substitute mechanism. The mutation of beta-catenin in the 3^rd^ exon could attract the target gene transcripts by Wnt signaling in the nucleus, resulting in the hyperpropagation even in the absence of APC mutation initiating tumorigenesis from adenoma to carcinoma pathway, implying the involvement of an alternative pathway in CRC tumorigenesis. The present data suggest that APC polymorphism preponderance can be varied based on the region and population. The genotypic frequencies of APC 1822 for AA, TA, and TT were seen to be 58.46%, 33.84%, and 07.69% in CRC patients; in controls, we observed the following genotyping frequency 69.23%, 24.61%, and 06.15%. This genotypic distribution pattern was seen according to the Hardy-Weinberg equilibrium with *P* < 0.001 and the A/T allele variant associated with CRC jeopardy. Thus, our results showed a significant association for the APC polymorphism in the studied population.

MTHFR polymorphisms are widely studied for many pathologies. Its involvement in CRC was associated with the fluctuations of folate levels mainly due to the 677 C4T and 1298 A4C variants. They are potentially known to cause cancer than their subsequent variation in the deoxynucleotide pool [54]. Deficiency of folate in tissues can cause deformed DNA synthesis in the rapidly replicating cells and reduce cell proliferation, altered morphology, and physiology of the cell. The effect of common MTHFR gene (C677T) polymorphism in CRC risk regarding the folate level is quite controversial. Still, global DNA hypomethylation and gene promoter hypermethylation are linked to the TT genotype, the MTHFR for declined levels of folate intake [55]. An association between MTHFR and CRC was seen in the cases of proximal tumor in female older candidates [56].

Similarly, a positive correlation was seen in the folate levels of TT genotype in the genomic DNA methylated leucocytes and transformed human lymphoblasts with no correlation for the wild-type MTHFR CC genotype [57, 58]. Conversely, it might be possible that the folates do not involve the propagation of CRC in the MTHFR 677 CC wild-type genotype. In agreement with the previous findings, we found an association between the CT + TT genotype and the frequency of CRC risking the entire patient's group compared with age-matched controls. However, the result was not statistically significant as we observed a significant difference in the distribution of the CT + TT genotypes. Shannon et al. [59] recommended that elevated risk of CRC is connected with the MTHFR TT genotype in older populations due to age-related disturbances of the folate metabolism. Previous studies reported association among the MTHFR C677T genotype, serum folate status, and hypermethylation of the promoter region in the three tumor-related genes (p16, hMLH1, and hMHSH2) among the CRC patients [60]. However, several studies observed positive associations between MTHFR 677TT genotypes and elevated CRC risk; in contrast, our results show statistical significance. Miao et al. [61] in China and Guerreiro et al. [62] in Portugal demonstrated that MTHFR 677TT is an aide of elevated CRC risk. Our analysis on MTHFR polymorphism showed deviation from the Hardy-Weinberg equilibrium in the genotype distribution in controls and exhibited insignificant results. Genotype distributions were in agreement among control with the Hardy-Weinberg equilibrium except for C677T MTHFR polymorphism. The frequency of MTHFR 677 CC, CT, and TT genotypes was seen to be 63.07%, 27.29%, and 09.32% in CRC patients and 72.30%, 23.07%, and 04.61% among controls. The genotype distribution pattern for 677 C-T followed Hardy-Weinberg equilibrium with a *P* value of 0.051 and suggested the C/T variant alleles be linked to CRC risk as the frequency of C677T polymorphisms was seen to be significant in our studies. Including more CRC cases can provide solid interpretations of the significance of the MTHFR and APC polymorphisms in the pathophysiology.

## 5. Conclusion

The present study suggests that progress in specialized transmission for detecting CRC could impact cancer mortality, thereby significantly improving public welfare. According to our current study, karyotyping the solid tumors will elaborate on the structural and chromosomal defects seen in the CRC pathophysiology, and we have observed the 5p region being constantly altered in CRC patients. Hence, karyotyping investigation regarding chromosomal aberrations in CRC aids support to assess organization and monitor treatment regimens. The present investigation revealed a significant association between CRC with APC and MTHFR polymorphisms, suggesting an approach to identifying the individual risk of CRC by assessing the genes for metabolizing enzymes and receptors.

## Figures and Tables

**Table 1:** Chromosomal aberration frequency in experiments based on their habits (smoker and alcohol).

S. no.	Particulars	Age (years) (mean ± SD)	No. of subjects	CTAs (mean ± SD)	CSAs (mean ± SD)	TCAs (mean ± SD)
1	CTL	54.63 ± 9.83 (36-75)	65	0.58 ± 0.55	0.36 ± 0.48	1.01 ± 0.85
2	CRC	54.72 ± 10.24 (37-78)	65	2.98 ± 1.11	2.38 ± 0.89	5.38 ± 1.84
3	Smoker (CRC)	57.70 ± 9.93 (38-78)	37	3.37 ± 1.06	2.54 ± 0.90^∗^	5.91 ± 1.78^∗^
	Nonsmoker (CRC)	50.92 ± 9.36 (37-67)	28	2.46 ± 0.96	2.17 ± 0.86	4.60 ± 1.68
4	Alcohol (CRC)	55.54 ± 11.05 (37-78)	43	3.10 ± 1.21^∗^	2.37 ± 0.92^∗^	5.48 ± 1.99^∗^
	Nonalcohol (CRC)	53.95 ± 8.00 (37-68)	22	2.68 ± 0.94	2.31 ± 0.89	4.95 ± 1.67

CTL: controls; CRC: colorectal cancer; smoker, nonsmoker, alcohol, and nonalcohol; CTAs: chromatid type aberrations; CSAs: chromosomal type aberrations; TCAs: total chromosomal aberrations. Values are presented as the mean ± SD. ^∗^Statistically significant compared to controls (*P* < 0.05).

**Table 2:** Chromosomal aberration frequency in experimental and control based on their age.

S. no.	Particulars	Age (years) (mean ± SD)	Group	No. of subjects	CTAs (mean ± SD)	CSAs (mean ± SD)	TCAs (mean ± SD)
1	CTL	42.62 ± 6.10 (36-49)	I	24	1.00 ± 0.00	0.50 ± 0.70	1.00 ± 0.00
		61.08 ± 6.32 (51-75)	II	41	1.05 ± 0.23	1.00 ± 0.00	1.59 ± 0.49
2	CRC	43.87 ± 3.62 (37-49)	I	24	1.83 ± 0.48	1.45 ± 0.50	3.29 ± 0.75
		62.79 ± 6.79 (51-78)	II	41	3.64 ± 0.78^∗^	2.91 ± 0.54^∗^	6.56 ± 1.06^∗^
3	Dukes stage A (CRC)	42.37 ± 3.62 (38-48)	I	8	1.75 ± 0.46	1.25 ± 0.46	3.0 ± 0.75
4	Dukes stage B (CRC)	44.62 ± 3.02 (37-49)	I	14	2.0 ± 0.00	1.5 ± 0.53	3.5 ± 0.53
5	Dukes stage C (CRC)	57.87 ± 5.59 (48-71)	II	25	3.5 ± 0.53	2.62 ± 0.91	6.12 ± 1.24
6	Dukes stage D (CRC)	64.87 ± 64.87 (57-78)	II	18	4.0 ± 0.75^∗^	3.12 ± 0.35^∗^	7.12 ± 0.83^∗^

CTL: controls; CRC: colorectal cancer; Dukes stage: A-D; group I < 50 and II > 50; CTAs: chromatid type aberrations; CSAs: chromosomal type aberrations; TCAs: total chromosomal aberrations. Values are presented as the mean ± SD. ^∗^Statistically significant compared to controls and within groups (*P* < 0.05).

**Table 3:** Cytogenetic patterns in CRC patients using GTG banding.

S. no.	Karyotype	Age (years) (mean ± SD)	No. of subjects	Chromosomes	Percentage
1	Deletion	53.94 ± 9.32	34	Chromosomes (5p, 9p, 13q, 16q, 19p, 18q)	52.30%
2	Duplication	55.47 ± 11.80	23	Chromosomes (7, 8q, 12p, 14q, 16q, 20q, 21q)	35.38%
3	Inversion	52.83 ± 10.04	6	Chromosomes (1q, 2p, 4p)	9.23%
4	Translocation	64.5 ± 2.12	2	Chromosomes (2, 11, 22)	3.07%

**Table 4:** Genotype frequency of *MTHFR* (C/T) and *APC* (A/V) in CRC patients and control.

Locus	Genotype	Patients (65)	Controls (65)	OR	95% CI	*χ* ^2^	*P* value
*MTHFR*	CC	41 (63.07%)	47 (72.30%)	0.57	0.26–1.26	1.41	0.235
	CT	18 (27.29%)	15 (23.07%)	0.97	0.65–4.10	2.73	0.001
	TT	06 (9.32%)	03 (4.61%)	2.49	0.61–10.10	0.98	0.322
*APC*	AA	38 (58.46%)	45 (69.23%)	0.68	0.28–1.27	1.28	0.257
	AV	22 (33.84%)	16 (24.61%)	1.65	0.78–3.49	1.28	0.257
	VV	05 (7.69%)	04 (6.15%)	1.96	0.58–8.75	0.78	0.426

Ile: isoleucine; Val: valine; OR: odds ratio; CI: confidence intervals; *P*: probability.

## Data Availability

The datasets generated during and/or analyzed during the current study are available from the corresponding author on reasonable request.
